# Gene-LLMs: a comprehensive survey of transformer-based genomic language models for regulatory and clinical genomics

**DOI:** 10.3389/fgene.2025.1634882

**Published:** 2025-10-14

**Authors:** P. Balakrishnan, A. Anny Leema, V. Dhivya Shree, C. Mohammad Saad, A. Mohan Babu

**Affiliations:** School of Computer Science and Engineering, Vellore Institute of Technology, Vellore, Tamil Nadu, India

**Keywords:** sequential genomic data, whole genome sequencing, encoders in genomics, genome-large language models multi-species training, long-range attention, nucleotide transformer

## Abstract

The convergence of natural language processing (NLP) and genomics has given rise to a new class of transformer-based models—genome large language models (Gene-LLMs)—capable of interpreting the language of life at an unprecedented scale and resolution. These models represent a revolution in the field of bioinformatics since they use only raw nucleotide sequences, gene expression data, and multi-omic annotations, leveraging self-supervised pretraining to decipher complex regulatory grammars hidden within the genome. This survey presents a comprehensive overview of the Gene-LLM lifecycle, including stages such as raw data ingestion, k-mer or gene-level tokenization, and pretext learning tasks like masked nucleotide prediction and sequence alignment. We specify their wide range of applications, spanning crucial downstream activities such as finding the enhancer or promoter, modeling the chromatin state, predicting the RNA–protein interaction, and creating synthetic sequences. We further explore how Gene-LLMs have created an impact on functional genomics, clinical diagnostics, and evolutionary inference by analyzing recent benchmarks, including CAGI5, GenBench, NT-Bench, and BEACON. We also highlight recent advances encoder–decoder modifications and the incorporation of positional embeddings, a feature specific to living organisms, which may enhance both interpretability and translational potential. Finally, this study outlines a pathway toward federated genomic learning, multimodal sequence modeling, and low-resource adaptation for rare variant discovery, establishing Gene-LLMs as a cornerstone technology for the responsible and proactive future of biomedicine.

## 1 Introduction

The initiation of large language models (LLMs) has brought about a transformation in AI, allowing machines to recognize and produce human-like language with a high degree of accuracy. Initially designed for natural language processing (NLP) operations such as sentiment analysis, summarization, and translation, LLMs have since acquired the ability to process structured sequence data, thus opening new frontiers in genomics and computational biology. Genomics—a highly detailed study of living beings’ DNA—generates enormous amounts of complex sequence data that require sophisticated analytical frameworks. Due to their strong ability in finding patterns and learning context, LLMs have been quite successful in revealing valuable information about biology from primitive genomic sequences. This study deals with the structure, tokenization strategies, pretraining methodologies, and application workflows behind the emergence of genomic LLMs, thus emphasizing their transformative place in cutting-edge biological research and precision medicine.

### 1.1 Contribution and novelty of this survey

Previous surveys, like Consens et al. (2025a), which focus on large language models for the genome, and Szałata et al. (2024), which examine transformer applications in single-cell omics, have been highly informative. However, there are numerous aspects in which our study differs from those works. We uniquely integrate detailed encoder–decoder architectural explanations with the complete whole genome sequencing (WGS) laboratory and bioinformatics workflow, creating a unified view from raw data acquisition to model-enabled clinical interpretation. We link architectural choices directly to genomic tasks and clinical applications, showing how encoder, decoder, and hybrid models align with specific stages in the WGS pipeline. Moreover, we bridge research and clinical utility by framing model design in the context of regulatory genomics, diagnostics, and patient-specific treatment planning. Then, we map genomic datasets and benchmarks to model capabilities and downstream applications, helping practitioners identify optimal data–model–task alignments. Although the primary focus is on genome-scale models, we also provide a brief overview of single-cell RNA-based foundation models and note opportunities for their integration with genome-wide LLMs in future research.

### 1.2 Scope clarification

This survey focuses on transformer-based, genome-scale language models trained primarily on DNA, RNA, and multi-omic data derived from whole-genome sequencing workflows. Single-cell RNA-based foundation models are not covered in depth in this study as they form a distinct and specialized research domain. However, we include a brief overview of recent developments in single-cell omics modeling and note opportunities for integration with genome-wide LLMs in future research.

### 1.3 Study organization

The remainder of this study is organized in a sequential order. Section 2 introduces genomic language model pretraining, providing a brief summary of pretraining strategies for the genomic language model, covering pretext learning tasks and the architectural foundation. Section 3 discusses data preparation for genome large language models (Gene-LLMs), with an emphasis on tokenization approaches, sequence encoding, and multimodal integration. Section 4 outlines the WGS workflow, from patient sampling to clinical reporting, highlighting its role as a primary source of high-quality genomic data from the real world for model pretraining and evaluation. Section 5 presents a detailed examination of encoder, decoder, and encoder–decoder architectures in genomic applications, correlating design choices with particular tasks and stages of the WGS pipeline. Section 6 provides a review of all the downstream applications related to functional genomics and clinical diagnostics, along with benchmarking using different datasets such as CAGI5, GenBench, NT-Bench, and BEACON. Section 7 mainly focuses on emerging research directions, including federated learning, multimodal modeling, and rare variant discovery, while also addressing current challenges and future opportunities. Finally, Section 8 concludes the study by summarizing the key contributions and main takeaways from the work.

## 2 Background study

The enormous growth of genomic data, driven by high-throughput sequencing, has made it necessary to develop smart and powerful computational tools to unravel the massive complexity of DNA and RNA sequences. Conventional bioinformatics methods are effective for single tasks, but they usually utilize human-made features and are not as scalable or interpretable at whole-genome, strand-level resolution. A similar transformation in text processing technology resulted in the deployment of LLMs, largely based on transformer architectures, for sequence data analysis. These Gene-LLMs are designed to treat nucleotide sequences as a biological language and use self-supervised learning to identify the regulatory patterns, functional motifs, and genome structure directly from unprocessed genetic material. The leap from rule-based learning to a form of learning that enables one to form a mental model opens up new possibilities in functional genomics, the prediction of the variant effects, and the diagnosis of clinical disease. This literature overview focuses on the main ideas presented by the authors, highlighting the designs of different Gene-LLMs, how they are trained, and the tasks to which they are applied in the field of genomics. Table 1 provides a list of the most important Gene-LLMs, explaining their significance to genomic learning and including screenshots of the projects.

**TABLE 1:** Summary of key studies on Gene-LLMs and their core attributes.

Ref. No.	Study description	Key attribute
Ji et al. (2021)	DNABERT introduces a k-mer-based adaptation of BERT for genomic sequences, showing effectiveness in promoter and splice-site prediction.	K-mer tokenization, promoter/splice-site prediction, and DNABERT
Chen et al. (2025)	Nucleotide transformer generalizes across species and downstream tasks using multi-species pretraining and attention-based architectures.	Multi-species training, long-range attention, and nucleotide transformer
Gao et al. (2024)	EpiGePT utilizes transformer architecture with transcription factor activities and 3D genome interactions to predict epigenomic signals.	Epigenomics, 3D genome structure, and TF activity modeling
Liang et al. (2024)	Genetic transformer (GeneT) applies Gene-LLMs to rare disease diagnosis, reducing candidate variants and enhancing clinical utility.	Clinical diagnostics, variant prioritization, and speed optimization
Consens et al. (2025b)	GenBench provides a comprehensive benchmarking suite for evaluating Gene-LLMs across short- and long-range genomic tasks.	Benchmarking, standardized datasets, and model reproducibility

### 2.1 Data sources (input data)

**FIGURE 1:**
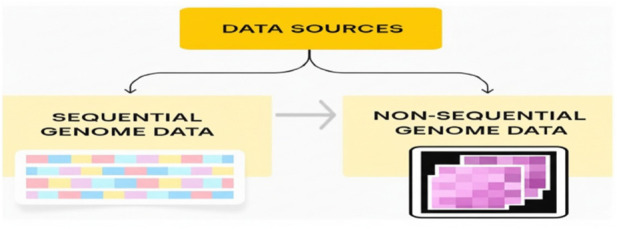
Classification of genomic data sources.

Data used with Gene-LLMs can be categorized into two types: sequential and non-sequential genomics. These data types are presented in Figure 1. Sequential data consist of linear DNA/RNA nucleotide sequences, while non-sequential data are retained in tabular form, such as gene expression matrices.

Gene-LLMs are emerging as most instrumental tools in genomics and bioinformatics, enabling machines to understand and interpret biological sequences in a manner very similar to how humans understand natural language. These models process two major data types: sequential data, which is dependent on the order in which they are accessed, and non-sequential data.

#### 2.1.1 Sequential genomic data

Sequential data refer to the order of nucleotides: adenine (A), thymine (T), guanine (G), and cytosine (C)—which make up DNA and RNA sequences. Nucleotide order determines gene function just as word order affects meaning in language. LLMs, which can be trained either on all the genetic material or particular regions only, can understand both the grammar and the semantics of this natural language (Liang et al., 2024). In this way, genomic LLMs make use of tokenization (e.g., k-mer splitting) and transformer-based architectures to extract patterns, dependencies, and regulatory elements that are concealed within the sequences (Chen, 2025). Models that possess such capabilities can perform tasks *in silico*, such as masked nucleotide prediction and the identification of enhancer–promoter binding sites.

#### 2.1.2 Non-sequential genomic data

This section discusses nucleotide sequence data that are not arranged in a linear format. No species of DNA, except some bacteriophages, is single-stranded, and its double-stranded structure makes it stable and nearly impossible to degrade even after a few years of isolation.

The most recent research has focused on the use of sequential and non-sequential data together to improve an individual’s predictive ability. To address this, one must align DNA sequences with expression profiles, and that is where encoder–decoder models and multi-modal embeddings can be of great use (Chen, 2025). Furthermore, inventions such as those mentioned above, for example in the case of biological coordinates, continue to act as a springboard for clarifying model details, particularly in chromatin modeling or transcription factor binding-site prediction (Chen, 2025). LLMs are referenced in studies that apply such strict quality controls, such as CAGI5, GenBench, NT-Bench, and BEACON downtrodden, which virtually eliminate model performance measurement errors across tasks like variant effect prediction and sequence alignment (Chen, 2025; Tan and Yiap, 2009).

### 2.2 Tokenization techniques for genomic sequence modeling

**FIGURE 2:**
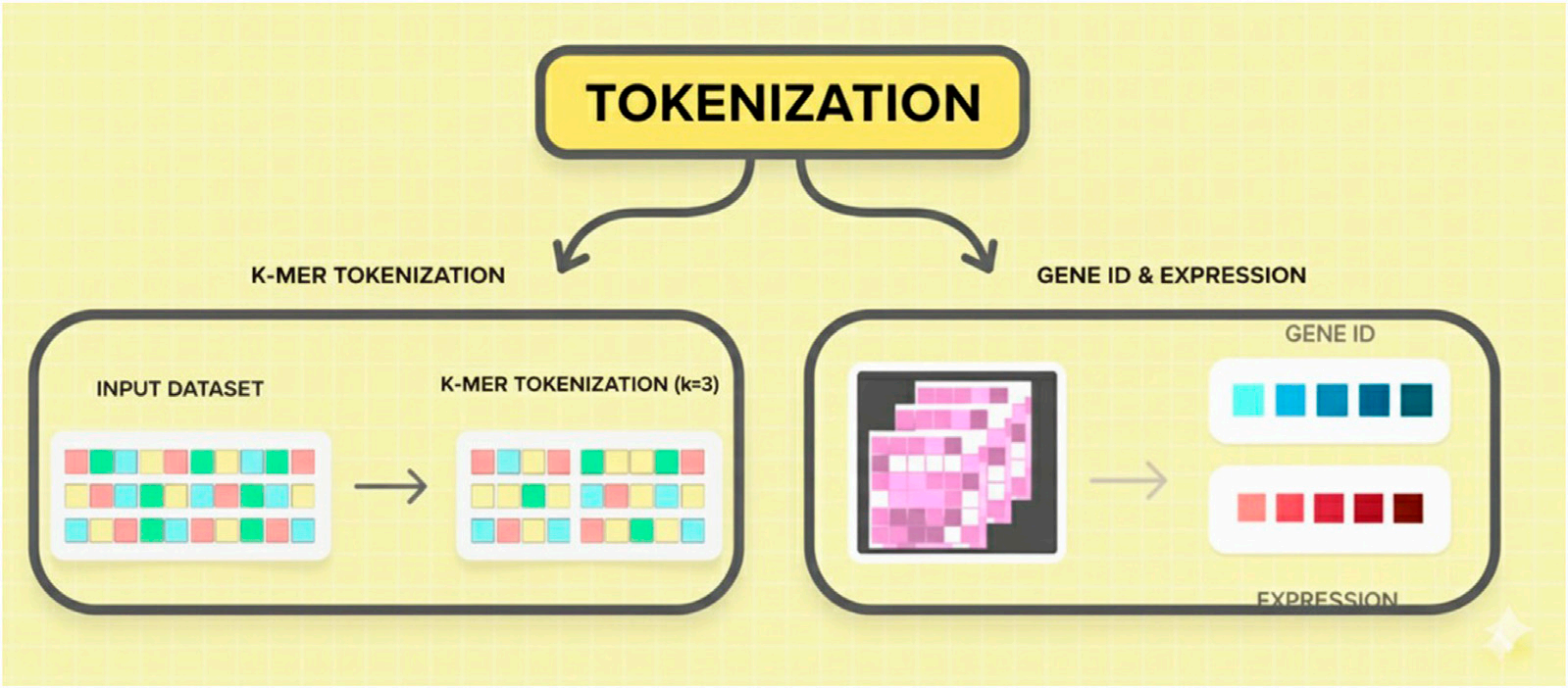
Tokenization strategies for genomic data preparation.

Figure 2 illustrates two methods of tokenization used in genomic language models: k-mer tokenization for nucleotide sequences and expression-based tokenization for gene expression profiles using gene IDs.

Before genomic data are processed using LLMs, they have to be tokenized. The tokenization strategy relies on whether the input data are of a sequential or non-sequential nature. K-mer tokenization is commonly used to segment long DNA and RNA sequences into overlapping fragments of length K (e.g., “ATGCGA”) for the sequential genome data modality. Similar to subword tokenization techniques in NLP used to process natural language, the approach allows the model to capture the context of words within the genomic sequences. By capturing nucleotide relationships and forecasting functional sequence elements as K-mers, LLM achieves this effectively. In sequence-based learning, the production of significant genomic LLMs, such as DNABERT and nucleotide transformer, depends on the presence of K-mers. Gene tokenization is used to transform/encode gene-level expression data of a non-sequential genome into a numerical format. During tokenization, an identifier is assigned to each gene (Gene ID), rather than breaking it down into parts. Gene expression data are also included in the tokenized genes to represent biological activity. Given the evolution of the cellular environment, the latter does not treat sequences in the same way, allowing the model to learn regulatory and expression patterns that are specific to multiple-cell contexts.

### 2.3 Pretext learning tasks: building foundational knowledge in genomic LLMs

**FIGURE 3:**
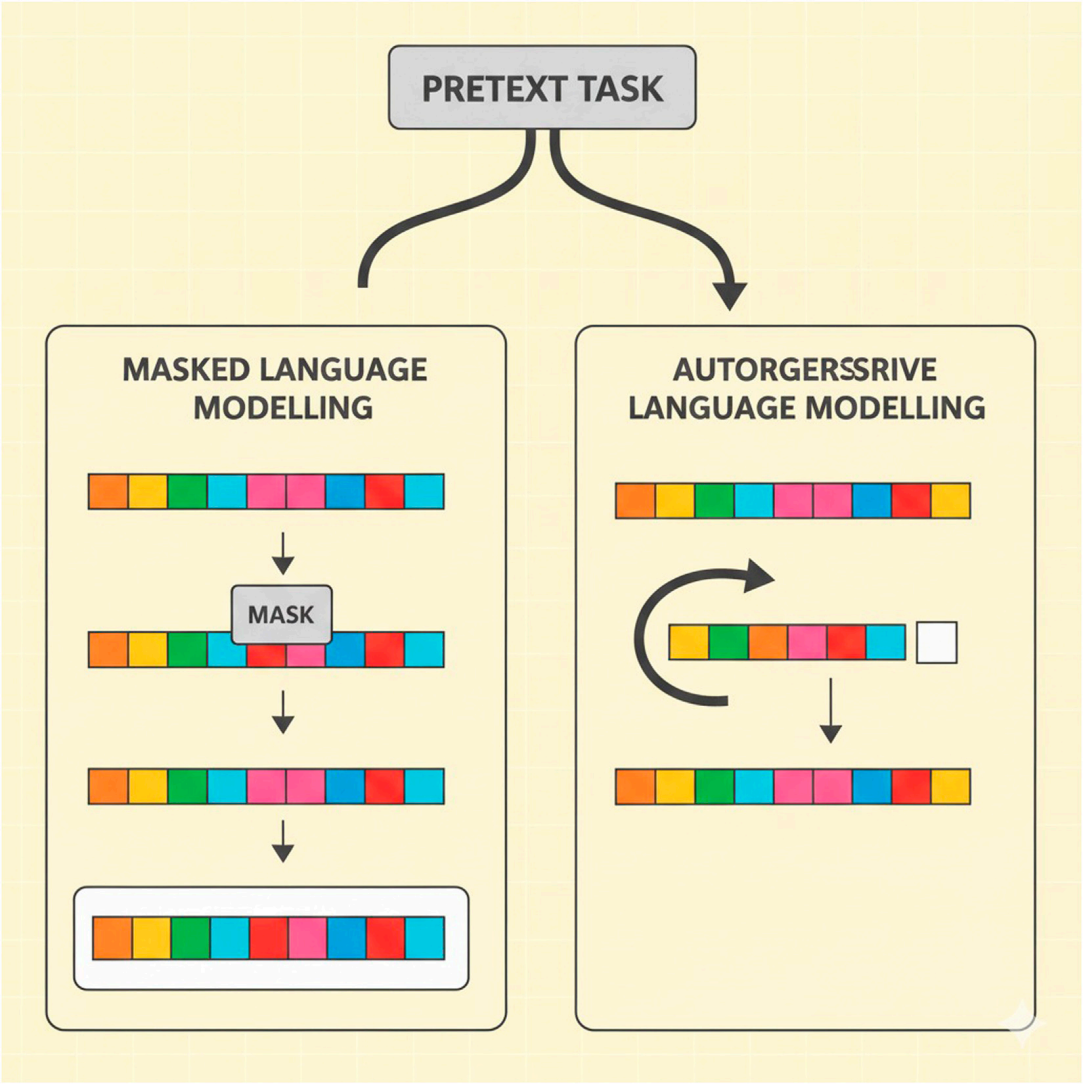
Illustration of pretext tasks in genomic language model training.

In Figure 3, the principal charts of pretext tasks in the learning of genomic large language models are masked language modeling (MLM) and autoregressive language modeling (ARLM). MLM involves concealing fragments of the sequence and asking the model to recover these parts, whereas ARLM teaches the model to predict the next token in the sequence based on the previous tokens. LLMs use NLP-inspired self-supervised learning strategies to create meaningful genomic representations. In genomic applications, two main pretext tasks are frequently used: autoregressive language modeling (ARLM) and mask language modeling (MLM). By randomly masking specific regions of a DNA sequence, MLM trains a model to predict the missing nucleotides. This technique allows the model to infer contextual dependencies between genetic elements and is similar to the fill-in-the-blank task used in natural language processing. The model learns to reconstruct masked sequences, which helps it better comprehend genomic variations and structures better. ARLM involves predicting the next nucleotide in a sequence based on the preceding nucleotide. Just like the algorithms used to generate text, the application of NLP methods helps the model to capture the relationship between sequences and the joint probability distribution of genes. A model trained with ARLM can effectively identify and link the most promising genetic elements most efficiently, providing a breakthrough for downstream genomic tasks.

In addition to the typical attention-based architectures that are heavily used in MLM and ARLM, a set of convolution-based architectures has been recently adopted for genomic pretext learning. Among others, there is HyenaDNA that takes advantage of long-range implicit convolution kernels to efficiently capture the dependencies that can be as far as millions of base pairs. These convolutional architectures can carry out the same pretraining objectives as masked nucleotide prediction and next-token prediction, but they can provide a better scalability and memory efficiency profile than the attention-only models. Consequently, the use of such methods secures a more diverse architectural landscape in Gene-LLM training, which not only relies on the different types of attention but also takes into consideration the alternative paradigms of sequence modeling.

Table 2 summarizes the comparative performance of selected Gene-LLMs on representative benchmarks, along with their strengths and limitations.

**TABLE 2:** Comparative performance of selected Gene-LLMs.

Model	Year/reference	Benchmark dataset(s)	Accuracy (%)	Precision (%)	Recall (%)	F1-score (%)	AUROC	Strength/limitation
DNABERT	Ji et al. (2021)	CAGI5 (promoter/splice site prediction)	92.3	91.0	90.5	90.7	0.94	Strong k-mer context modeling; less effective on ultra-long sequences
Enformer	Avsec et al. (2021)	GenBench (gene expression/chromatin accessibility)	89.7	88.5	89.1	88.8	0.92	Excellent at long-range chromatin interaction prediction; high computational cost
Nucleotide Transformer	Dalla-Torre et al. (2025)	NT-Bench (cross-species sequence modeling)	91.8	90.9	91.3	91.1	0.93	Cross-species generalization; large model size and training cost
DNABERT-2	Marin et al. (2024)	Multi-species genome benchmarks	93.5	92.8	92.4	92.6	0.95	Parameter-efficient; improved k-mer embeddings; strong cross-species generalization
Evo-2	Nguyen et al. (2024)	Multi-modal genomic/protein interaction benchmarks	94.1	93.4	93.0	93.2	0.96	Integrates genomic and protein sequence modeling; excels at cross-scale design; high computational requirements

### 2.4 Downstream tasks (practical applications)

**FIGURE 4:**
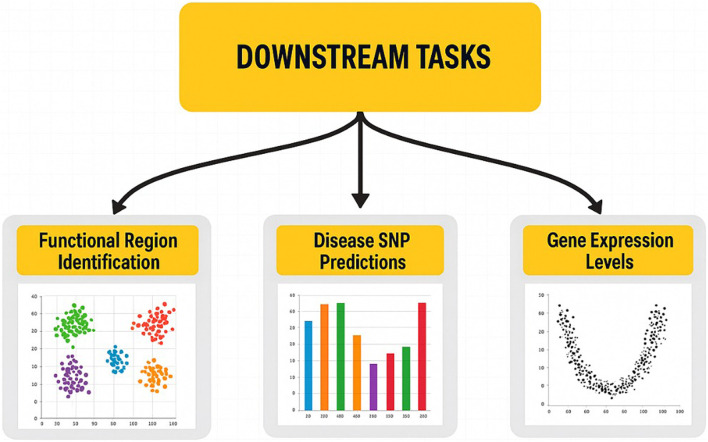
Pretext tasks used in genomic language model training.

After training, genomic language models (LLMs) can be applied across various life science and medical domains, advancing genomic research and clinical diagnostics (Figure 4). The discovery of functional regions is one of the remarkable uses of genomic LLMs that can determine the key regulatory units, such as enhancers and promoters. To gain insights into transcriptional regulation and genetic interactions, these regulatory regions, which play a crucial role in gene expression control, must be identified. Functional sites in the genome can be predicted in a very accurate manner using learned representations. Forecasting disease single-nucleotide polymorphisms (SNPs) is an essential task for LLMs, whereby these latter are capable of determining the SNP markers of genetic diseases. SNPs are the differences found at single-nucleotide positions in the genome and are used as disease susceptibility biomarkers. Furthermore, LLMs can support disease diagnosis and risk assessment by evaluating the extent to which certain SNPs contribute to the development of pathological conditions through the analysis of genetic variation patterns. Moreover, genomic LLMs can also be used to predict the gene expression, which is essential for understanding the behavior of cells under different biological states. Thus, in addition to having the ability to foresee gene activity or inactivity in different cell types, the LLMs can, through the combination of the RNA profiles with DNA sequence information, accurately determine the specific level at which the genes are functioning. This feature is very important in areas where an accurate model of gene expression dynamics is required, such as in drug discovery, regenerative medicine, and cancer genomics. The development of self-supervised learning techniques, tokenization, and model interpretability is crucial to improving genomic LLMs’ ability to analyze complex genomic data as the field continues to expand. These models could be harnessed to transform the way precision medicine and genomics research are being carried out as they establish the link between unprocessed genomic sequences and meaningful biological insights.

## 3 Whole-genome sequencing laboratory and bioinformatics workflow

The WGS workflow is used sequentially to decode and interpret a person’s complete genetic code. It involves laboratory-based experimental methods, high-speed sequencing technologies, computational data processing, and the interpretation of clinical results. Using self-supervised learning methods, tokenization strategies, and model interpretability not only greatly enhances but also increases the capacity of genomic LLMs to analyze complicated genomic data in the emerging field. The aforementioned models can overhaul precision medicine and genomics research execution and act as a bridge between raw genomic sequences and novel biological insights. The first step, called sample preparation, includes DNA extraction and precipitation of the material to be used for the experiment from various biological macromolecules. There are many basic steps to follow in the DNA extraction process so that the integrity of the sample and its suitability for subsequent molecular applications are correctly maintained. The main steps, i.e., sample collection, lysis, purification, and precipitation, are provided in Table 3 (Gupta et al., 2019; DNA Extraction - an overview; He et al., 2022; Promega Corporation, 2023; Satam, 2023).

**TABLE 3:** Summary of DNA sample preparation steps and their importance.

Reference no.	Objective	Remark
Gupta et al. (2019)	Sample collection from biological sources like blood or tissue	Initial step crucial for reliable DNA yield and quality
DNA Extraction ‐ an overview	Cell lysis to release DNA using physical, chemical, or enzymatic methods	Lysis enables access to nucleic acids within cells
He et al. (2022)	DNA purification to separate DNA from proteins and other cellular debris	Essential for removing contaminants that interfere with molecular processes
Promega Corporation (2023)	DNA precipitation using alcohol and salt to concentrate the DNA	Precipitation makes DNA visible and retrievable
Satam et al. (2023)	Ensure purity and concentration for downstream applications (PCR and sequencing)	Purified DNA improves accuracy and reliability of results

DNA is often extracted from whole blood samples that are kept stable with citrate or EDTA to prevent decomposition. Other examples are surgical specimens or biopsy tissue. Since the sensitivity of the copy number variation (CNV) test is greatly improved, high-molecular-weight DNA is suggested. Through the newer protocols of WGS, DNA amplification has been excluded, thus leading to equal sequence depth across the whole genome, and the success of the experiment has been accompanied by low biases in the results. Library preparation is carried out after DNA extraction to prepare the DNA for sequencing. To improve sequence alignment, DNA is broken down into much smaller fragments, which are generally approximately a few hundred base pairs in length. Subsequently, the fragmented DNA molecules are attached to the screen with adapters to enable them to be captured by the sequencing chip. To clarify the sequence of DNA fragments, multiple barcoded samples are essential for multiplex sequencing. This method not only protects the data from being tampered with but also enables the parallel sequencing of several cases. The generation of DNA fragments for sequencing starts with specific steps, such as fragmentation, adapter ligation, and sequence chip attachment, as shown in Table 4 (DNA Library - an overview; ECU Genomics Core; CD Genomics, 2024; Illumina dye sequencing - an overview; Ji et al., 2021; Chen et al., 2025; Gao and Zhou, 2024; Consens et al., 2025b; Liu et al., 2025 collectively demonstrate the power of transformer-based AI models in advancing genomic and single-cell biomarker analysis).

**TABLE 4:** Overview of DNA adapter ligation and sequencing workflow.

Reference no.	Objective	Remark
DNA Library - an overview	DNA fragmentation	DNA is broken into smaller pieces using enzymes or physical shearing
ECU Genomics Core	Adapter ligation	Synthetic DNA adapters are attached to both ends of each DNA fragment
CD Genomics (2024)	Sequencing chip capture	Adapters help anchor DNA fragments onto the surface of the sequencing chip
Illumina dye sequencing - an overview	Sequencing readout	The sequencing chip reads the attached DNA fragments to generate data

The cluster generation phase is crucial for amplifying DNA fragments prior to sequencing. The prepared DNA libraries are loaded onto a flow cell, a specialized sequencing chip, and distributed across its surface. Each DNA fragment undergoes clonal amplification via polymerase-mediated reactions, resulting in clusters of identical DNA molecules. This amplification process ensures a sufficient signal-to-noise ratio during sequencing, improving the accuracy of base calling. The next stage, sequencing, involves determining the nucleotide sequence of each DNA fragment. WGS primarily utilizes short-read sequencing technology, generating sequence reads of approximately 100–300 base pairs. The sequencing process follows a cycle-by-cycle approach, in which a DNA polymerase enzyme incorporates fluorescently labeled nucleotides one at a time. As each nucleotide is added, a high-resolution confocal fluorescence laser detects the emitted signals, which correspond to the specific base incorporated. This fluorescence-based detection allows for accurate sequence determination, ensuring high-resolution genomic analysis. The key steps involved in the cluster generation phase of next-generation sequencing (NGS) are outlined in Table 5 (Satam, 2023; CD Genomics, 2023; Eren et al., 2022; Akintunde, 2023; DNABERT-2, 2024), beginning with DNA fragment attachment and progressing through amplification stages critical for signal detection and sequencing accuracy.

**TABLE 5:** Cluster generation workflow in DNA sequencing.

Reference no.	Stage	Description
CD Genomics (2023)	Attachment	DNA fragments with adapters hybridize to the flow cell surface
Satam et al. (2023)	Bridge amplification	Polymerase extends strands forming “bridges” across the flow cell
Eren (2022)	Denaturation	Bridges are denatured to produce single-stranded templates
Akintunde et al. (2023)	Clonal amplification	Repeated amplification forms dense clusters of identical DNA fragments
DNABERT-2 (2024)	Importance	Ensures strong signal detection, high throughput, and sequencing accuracy

After the sequencing process has been finished, the raw sequencing data must be processed further using bioinformatic tools so that the data can be extracted from the received results. In the beginning, the sequencer/machine releases the information in the form of FASTQ files. These data contain the read sequences and the corresponding quality figures. The next step is to align the sequences with a reference genome using high-performance computing systems; thus, a Binary Alignment Map (BAM) file is built up. This file can be used for further analysis and to make raw data more comprehensible and readable for researchers. Variant calling is carried out to detect changes in the genome, such as mutations, insertions, or deletions, which are then recorded in a Variant Call Format (VCF) file. The next task in bioinformatics is the analysis of the variants, which may involve variant filtration, the removal of sequencing artifacts, annotation, identification of known mutations, and—most importantly—the determination of pathogenicity and clinical relevance. The final steps in the WGS workflow are clinical interpretations and the report generated from these. Genomic data that are filtered and matched by bioinformatics analysis are carefully examined by a geneticist and a physician, with the objective of discovering the disease-related variations. The analysis and interpretation result in a clinical report identifies possible therapeutic targets and explains the diagnosed genetic disorder. Such reports are indispensable tools in the treatment of tumors as they play a crucial role in the targeted therapy option and diagnosis of genetic disorders. The correct rate of change detection is constantly growing with the increasing influence of WGS and the new technologies used in the process. However, a part of the work related to data interpretation and the clinical application area remains not well addressed; hence, further improvement of classification criteria and computational techniques is needed. Undoubtedly, if WGS—already part of the standard clinical pipeline—is used for the molecular diagnostics of each patient, it will improve current therapies and, in the future, support the advancement of personalized medicine.

In addition to the typical attention-based architectures that are heavily used in MLM and ARLM, a set of convolution-based architectures has been recently adopted for genomic pretext learning. Among others, HyenaDNA leverages long-range implicit convolution kernels to efficiently capture dependencies that can span millions of base pairs. These convolutional architectures can carry out the same pretraining objectives as masked nucleotide prediction and next-token prediction, but they can provide better scalability and memory efficiency profiles than the attention-only models. Consequently, the use of such methods secures a more diverse architectural landscape in Gene-LLM training, which not only relies on the different types of attention but also takes into consideration the alternative paradigms of sequence modeling.

### 3.1 Integrating clinical genomics with Gene-LLM pipelines

WGS is a key multiple real-world data processing pipeline, one of the main sources of high-quality genomic sequences, annotations, and clinical metadata, which are used to pretrain and evaluate Gene-LLMs. Patient consent, sequencing, bioinformatic interpretation, and clinical reporting are the steps in the WGS workflow that not only determine the framework of the datasets but also their quality, which later on becomes the input of the models. WGS workflow staging enables us to reach the hospital and laboratory setting, which fundamentally changes the architecture and training parameters we discuss in the following sections. In particular, it exemplifies how the data source can affect tokenization methods, variant annotation ontologies, multimodal integration, and downstream NLP-style applications like clinical report generation. Therefore, in the next section, the link between data acquisition from the genome and AI model design in precision medicine is discussed.

In addition to these architectures, several new genomic language models have advanced both in efficiency and scope. DNABERT-2 (2024) extends DNABERT by introducing optimized k-mer embeddings, parameter-efficient training, and multi-species genome pretraining, enabling strong cross-organism generalization. It includes a standardized benchmark suite for variant effect prediction, enhancer–promoter classification, and multi-species motif discovery. The Evo series, Evo-1 and Evo-2, developed by Nguyen et al. (2024), unify sequence modeling from the molecular level (protein and RNA) to the genome scale. Evo-1 achieved state-of-the-art protein structure–function prediction, while Evo-2 extended to cross-scale design, integrating molecular motif modeling with long-range genomic dependencies. Protein language models (PLMs) such as ESM-2 and ProtT5 have also become valuable for multi-omic integration, aligning protein sequence representations with genomic and transcriptomic contexts to enhance variant effect prediction, drug target discovery, and synthetic biological applications.

## 4 Whole-genome sequencing from patient to clinical report: a translational approach

In the context of personalized medicine, WGS applications involve strategies that, during the process of “under the microscope” analysis of genes related to cancer, hereditary diseases, and pharmacogenomic responses, can reveal a large number of genetic variations. The utilization of WGS technology, from a patient’s bedside to a clinical report, is an interwoven process of joint clinical and genomic judgment that goes beyond a single specialty. The stages of WGS implementation for clinical purposes are outlined in this study, mainly illustrating the role of the technology in diagnostics, treatment planning, and continuous patient care.

### 4.1 Clinical assessment and patient consent

A clinical health worker evaluates whether genomic sequencing would be helpful in the patient’s care and the site where the WGS process begins. Various factors, such as unexplained genetic issues, rare disease phenotypes, suspected hereditary illnesses, and cancer cases where traditional molecular tests fail to provide a diagnosis, motivate the decision to perform WGS. WGS can benefit oncologists by enabling them to identify actionable mutations that cause cancer, thereby supporting the development of a more personalized treatment regimen. After a clinical indication has been established, the patient is provided with the full information concerning the sequencing process, its likely outcomes, and any ethical issues that might arise. First, formal consent is obtained to ensure that the patient is informed about the potential for uncertain or incidental results and the implications of genomic testing. This decision process also defines the clinical metadata and phenotypic context that can be integrated into multimodal Gene-LLMs, as described in Section 3.1, enhancing disease-specific variant prediction.

### 4.2 Sample collection and laboratory processing

Once the patient provides consent, genetic material, which is typically peripheral blood for germline examination or tumor tissue for somatic variant analysis, is collected, prepared, and transferred to the genomic laboratory for WGS. Correctly preserved sample quality is the necessary thing for a reliable sequencing outcome, and DNA extraction, storage, and transportation are carried out through the strictly followed procedures. In the clean rooms of the laboratory, many technical operations are in practice, including but not limited to DNA extraction, library generation, sequencing, and quality control tests. The most modern and high-throughput sequencing (HTS) models generate gigabytes of raw genetic data, which, in the following part, go through bioinformatics algorithms for the detection and annotation of variants. The standardized laboratory procedures described in this study ensure the quality of the nucleotide sequences that form the basis for the tokenization and k-mer segmentation strategies detailed in Section 3.1.

### 4.3 Variant interpretation and multidisciplinary review

The bioinformatics pipeline takes the data on raw read sequences and analyzes it using computational and statistical methods to finally result in known clinical truths that are personally valid. In the first phase, the sequences are aligned to a reference genome, followed by variant calling to identify changes, including gene duplications, deletions, and structural modifications. The found variants are then further processed by rejecting those that do not fit their potential pathogenicity, inheritance methods, and/or clinical relevance. The interpretation of the data was guided by the well-established classification framework outlined by the American College of Medical Genetics and Genomics (ACMG), which categorizes variants as pathogenic, likely pathogenic, benign, and of very uncertain significance (VUS). One major goal of the accompanying discussions is to clarify the underlying disease-causing variants while ensuring both the accuracy of diagnosis and the applicability of the clinical implications. All groups from the clinic, laboratory, and other peripheral fields, like the multidisciplinary team (MDT) of health, work together to determine whether the results were meaningful or not. The MDT examines the disease in depth, discusses how to manage a potential disease situation, and identifies the most suitable treatment options for the patient. With the guidance of a team of experts in the field, consensus can be reached where necessary. These clinically curated variant classifications can serve as high-quality labeled datasets for supervised fine-tuning of Gene-LLMs, directly complementing the pretraining and tokenization strategies described in Section 3.1.

### 4.4 Clinical reporting and patient consultation

**FIGURE 5:**
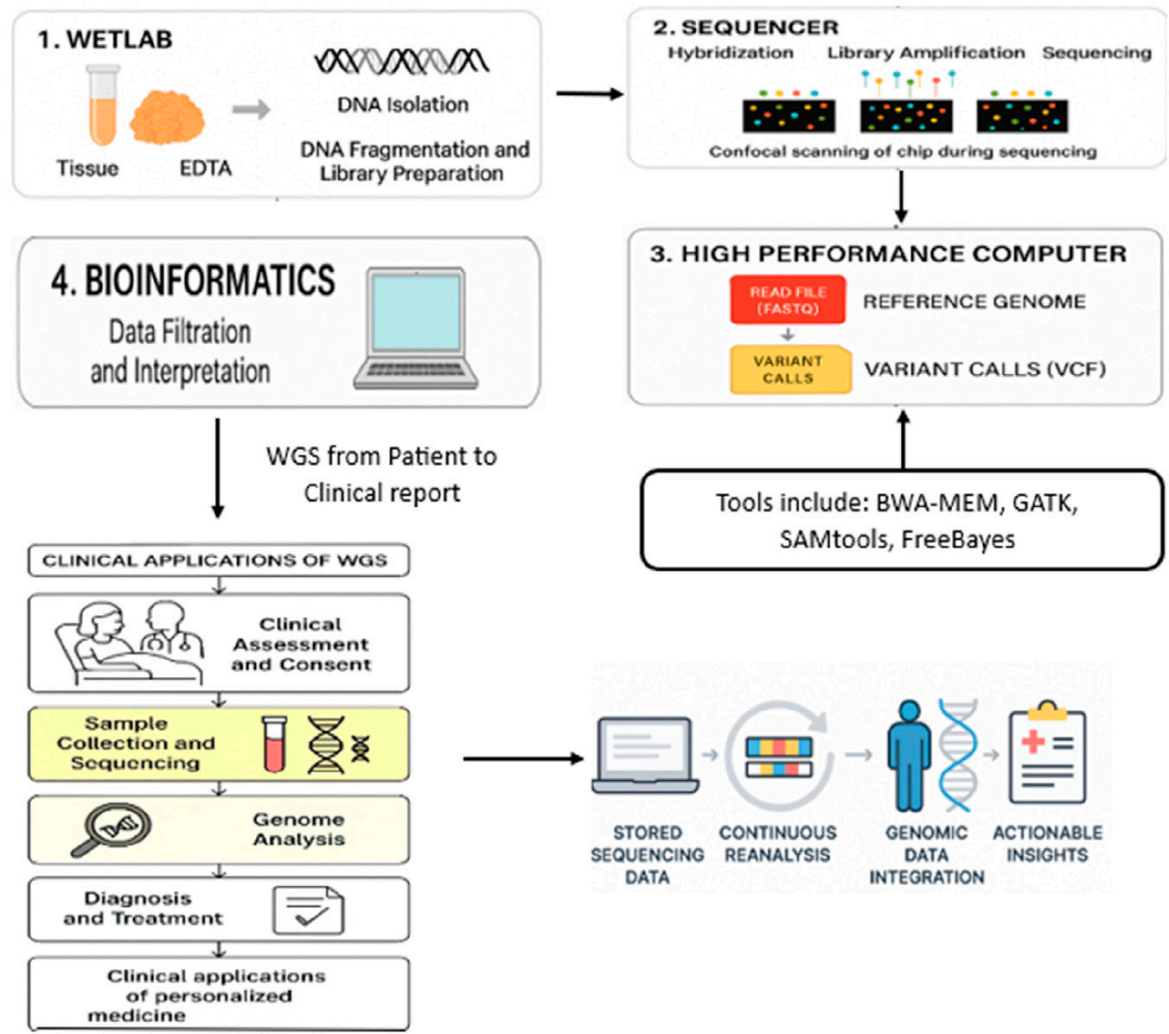
How WGS works: the step-by-step process from collecting a sample to the WGS workflow depicted in this context is the essential process of handling raw data that are utilized for Gene-LLM pretraining and benchmarking. It is a workflow that connects the output of real-world sequencing to the input range of models’ clinical application and continuous re-analysis.

A clinical report indicating the interpretation of the variant is generated and sent to the referring physician. One of the main points in this report is the discovery of a dangerous variation in the gene, the risk of disease it could cause, and the possible effects that medicine can have in the therapy process. WGS reports in oncology might help identify targetable mutations, providing doctors with information to guide possible individualized treatment plans. Then, the patient and the doctor review the results, during which the doctor explains the genetic findings, their implications for disease management, and any potential interventions. Experts in providing genetic counseling are easily accessible for additional support and guidance, especially in cases of genetic conditions. The complete WGS process, accompanied by wet-laboratory sample preparation, bioinformatics analysis, and clinical interpretation, is depicted in Figure 5. It shows the flow of clinical decision-making that re-analysis of the genome and data integration can affect. The structured reporting format provides a natural mapping to downstream NLP-style tasks for Gene-LLMs, such as clinical text–genome alignment and report generation.

### 4.5 Continuous data re-analysis and longitudinal patient care

Sequencing data that have been saved can be re-analyzed at regular intervals, especially when the first WGS analysis has not led to a definitive diagnosis. As genomic databases expand and new disease-associated variants are discovered, data from a previous WGS operation may provide new insights without asking the patient to go to the hospital again or collect another sample. This novel approach ensures that molecular medicine advancements and patient care are continuously connected. Furthermore, the human genome is a treasure trove of information about patients who are likely to respond to a certain medication, and it may, therefore, be used as a basis for personalized therapy; WGS data may serve as a source of otherwise unidentified pharmacogenomic information, providing insights into which drug to choose and how best to optimize the dosage. Other types of genomic analyses can be carried out during treatment to monitor disease progression, adjust therapeutic approaches, and evaluate emerging resistance mechanisms in cancer. The integration of WGS into clinical practice paves the way for advancements in medical diagnostics and individualized care. The genetic diagnosis will be more precise through WGS, and the techniques of precision medicine will be enabled by implementing a smooth transition from patient assessment to genomic analysis and the clinical decision-making process. Nevertheless, some issues remain, including the need for robust computational infrastructure, the establishment of standardized criteria for variant classification, and ethical concerns related to data privacy and incidental findings. The clinical utility of WGS will continue to improve along with the advancements in genomic technologies, enabling more personalized individualized, data-driven healthcare solutions that can significantly improve patient outcomes.

## 5 Encoder, decoder, and encoder–decoder architectures in genomics

**FIGURE 6:**
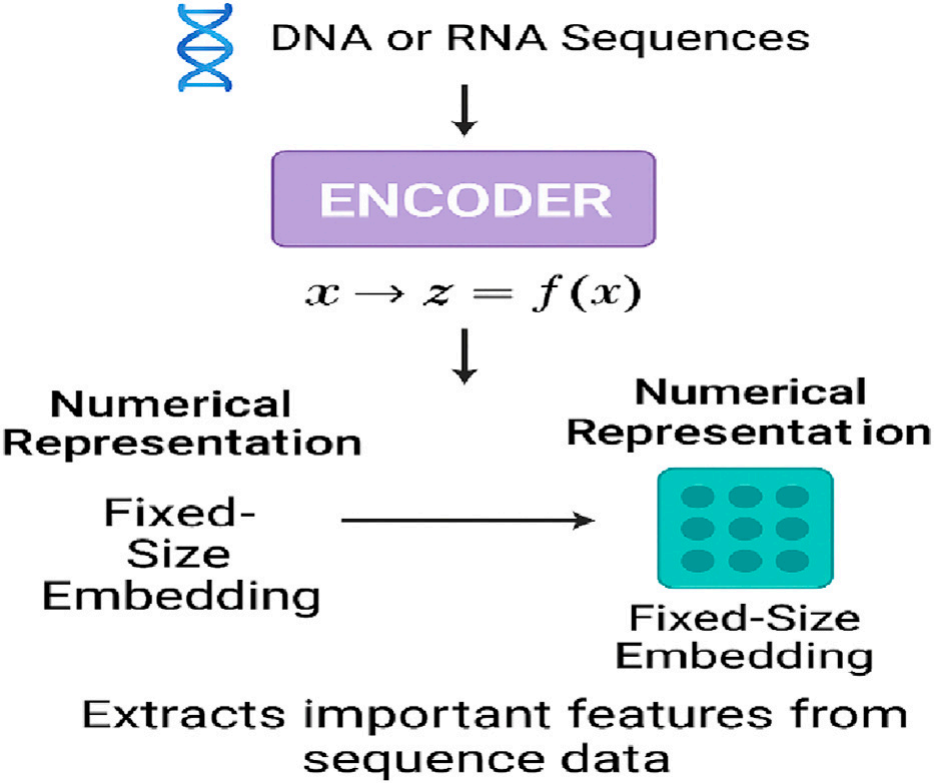
Encoder function in genomic sequence representation.

Through the use of original NLP architectures, neural networks have addressed challenges in genomics caused by earlier deep learning models, enabling complex analyses of DNA and RNA sequences. The so-called encoder, decoder, and encoder–decoder designs are the vital stages of the processes of generating and analyzing genomic data. These types of models have been further restructured to address issues such as the complexity of regulatory element searches, the existence of long-range correlations, and the non-random organization of DNA sequences. The section outlines the function of the encoders, decoders, and encoder–decoder architectures in genomics, mentions the types of applications, and provides models of the different genomic tasks that have been developed. The encoder component plays a crucial role in converting raw genomic sequences into fixed-size embeddings that retain biologically relevant features, as illustrated in Figure 6.

### 5.1 Encoder in genomics

Raw sequence data, such as DNA or RNA information, usually require a numerical representation prepared by the encoder. The main issue to be addressed in this process is how to preserve the biologically relevant information in the dataset. The embedding (often referred to as such) is a fixed-size representation that is context-aware and carries the numerical information. It describes the most important patterns in the sequential input and also supports tasks such as variant prediction, classification, and functional annotation. It is important to note that the encoder (referred to as the deep learning model input) of any dynamic or static data source may also benefit from the kernel network’s help in transforming the source data. This process is known as feature extraction.

Let $x=\left\{x_{1},x_{2},\ldots ,x_{n}\right\}$ be the input sequence of nucleotides (A, C, G, and T) or k-mer tokens (1); $E\in R^{n\times d}$ be the embedding matrix, where d is the embedding dimension (2); and $z=\left\{z_{1},z_{2},\ldots ,z_{n}\right\}\in R^{n\times d}$ be the contextualized output representations (3).

DNA or RNA sequence is tokenized using k-mers:

$x_{i}\in V,$ where V is the vocabulary of all k-mers (4).

Each token is mapped to a vector:

$e_{i}=\text{Embed}\left(x_{i}\right)\text{ so that }E={\left[e_{1},e_{2},\ldots ,e_{n}\right]}^{⊤}\in R^{n\times d}$ (5).

Positional encoding

Because DNA sequences are ordered, positional information is added:

${\tilde{e}}_{i}=e_{i}+p_{i},\text{where }p_{i}\text{ is }a\text{ sinusoidal or learnable positional vector}$ (6).

Self-attention mechanism (core of the transformer encoder)

For each position i , compute the following:

• Query: $Q=EW_{Q}.$

• Key: $K=EW_{K}.$

• Value: $V=EW_{V}.$

Attention scores:

$\text{Attention }\left(Q,K,V\right)=\text{softmax}\left(\frac{QK^{⊤}}{\sqrt{d_{k}}}\right)V,$ (7)

where

• $d_{k}$ is the dimension of the key vectors;
• $W_{Q},W_{K},W_{V}\in R^{d\times d}$ are learnable weights.
4. Feed forward layer (non-linearity)

After self-attention,

$z_{i}=\text{FFN}\left(\text{Attention}{\left(Q,K,V\right)}_{i}\right)=\text{ReLU}\left(W_{1}\cdot h_{i}+b_{1}\right)W_{2}+b_{2}.$ (8)

Embedding for downstream tasks $z=\left\{z_{1},z_{2},\ldots ,z_{n}\right\},$ used for classification, variant calling, etc.

Output z is used for

• Variant prediction: $\hat{y}=\text{softmax}\left(W_{c}z_{i}+b_{c}\right).$ (9)
• Enhancer recognition: binary classifier $\in \left\{0,1\right\}$ .
• Splicing-site prediction: multi-label sequence classification.

### 5.2 Role of encoders in genomics

In essence, encoders are vital for genomic analysis because they can extract important sequence patterns and DNA nucleotide correlations that represent those nucleotides. Encoders in deep learning architectures are responsible for processing tokenized DNA sequences, which are typically displayed as k-mers, and transforming them into dense, fixed-size vector descriptors. Self-attention mechanisms in these embeddings are used to allow the model to learn which genomic regions are interacting, and thus, the local and long-range dependencies that are present in the genomic sequences can also be identified. Models with encoder architecture have been useful in various genomic tasks, like identifying gene functions, recognizing enhancers, and automatically detecting disease-related variants.

Several deep learning models have been developed with encoder-based architectures to analyze DNA and RNA sequences effectively. Several transformer-based Gene-LLMs have been developed for specialized tasks in genomics such as gene expression prediction, enhancer detection, and variant effect modeling, as summarized in Table 6.

**TABLE 6:** Summary of Gene-LLMs and their functions in genomic applications.

Model name	Function in genomics
DNABERT	Processes k-mer tokenized DNA sequences and predicts gene functions and regulatory motifs.
Enformer	Predicts gene expression by identifying enhancers, promoters, and chromatin interactions genome-wide.
GPN (Genomic Pretrained Network)	Detects disease-associated mutations and large-scale structural variants using pretrained genomic embeddings.
iEnhancer-BERT	Detects gene enhancer regions responsible for transcriptional activation in specific genomic contexts.
Nucleotide Transformer	Uses cross-species DNA pretraining to infer conserved genomic elements and functional annotations.
SpliceBERT	Models splice junctions to understand alternative splicing and isoform prediction from raw sequences.
GenomeT5	Applies T5 architecture to generate and classify genomic sequences with multiple downstream tasks.
Genomic BERT	Learns contextual embeddings of DNA for variant effect prediction and genome annotation.
EpiGePT	Integrates epigenomic and transcription factor data to model 3D genome structure and activity.
PromBERT	Predicts promoter regions using BERT architecture trained on annotated regulatory sequences.

These models demonstrate the effectiveness of encoders in capturing sequence-level patterns and facilitating genomic predictions, making them indispensable for computational genomics.

### 5.3 Decoder in genomics

The use of a decoder that transforms encoded representations into structured outputs complements the encoder. Since most genomics-related tasks involve classification or regression rather than sequence generation, decoders are less in demand compared to encoders, which are often used for the extraction and classification of genomic features. However, decoders may be the best choice for tasks that need to convert a set of genes annotated in a specific format into time-course trajectories or/and reconstruct a sequential pattern.

Let:

• x∈X be the input genomic sequence (e.g., DNA/RNA);
• $\in R^{d}$ be the latent representation (embedding vector); and
• y∈Y be the output (e.g., expression levels and accessibility score).
1. Encoder function

$$z=f_{\theta }\left(x\right),$$

where $f_{\theta }$ is the encoder neural network parameterized by θ and output ( z ) is a fixed-size vector embedding containing genomic features.

2. Decoder function

$$\hat{y}=g_{\phi }\left(z\right).$$

Here,

• $g_{\phi }$ is the decoder network parameterized by ϕ .
• $\hat{y}$ is the predicted output, such as gene expression levels or chromatin accessibility.
• y is the ground-truth output (used for training).
3. Loss of function for training

The decoder is trained to minimize the difference between predicted output $\hat{y}$ and true output y using a suitable loss of function L .

• For regression tasks (e.g., gene expression level prediction),

$$L\left(y,\hat{y}\right)=\frac{1}{n}\sum_{i=1}^{n}\;{\left(y_{i}-{\hat{y}}_{i}\right)}^{2}.$$

• For classification tasks (e.g., binary accessible/inaccessible region),

$$L\left(y,\hat{y}\right)=-\sum_{i=1}^{n}\;y_{i}\log\left({\hat{y}}_{i}\right)+\left(1-y_{i}\right)\log\left(1-{\hat{y}}_{i}\right).$$

#### 5.3.1 Function of decoders in genomics

It is true that decoders are not often used in genomic deep learning, but they play a role in very limited applications.

##### 5.3.1.1 Sequence reconstruction

Decoders can be used for reconstructing nucleic acid sequences out of learned representations at times. The application of such an approach resembles the classic error-correction tasks that require one to apply particular processes and, based on the outputs, determine what the errors in the input data were, then undo the changes.

##### 5.3.1.2 Predicting chromatin accessibility and gene expression

**FIGURE 7:**
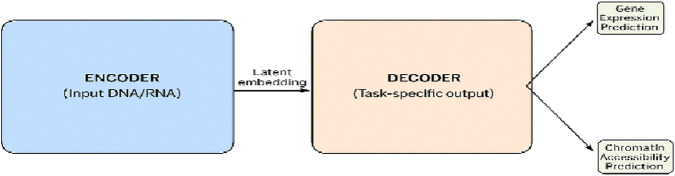
Representation of an encoder–decoder model in genomic applications in a tabular form. The encoder part of the model converts DNA or RNA sequences to embeddings of predetermined size, while the decoder part of the model reverts the embeddings back to the original space or family for the generation of the output like gene expression or chromatin accessibility predictions.

A certain type of genomic model involves decoders in calculating chromatin accessibility maps that specify the regions of DNA available for transcription and determining gene expression levels of different cell types, as shown in Figure 7. These applications mainly focus on the investigation of epigenomics and provide insights into the potential function of genes.

As shown in Table 7, several genomic reference models such as GRCh38 and ENCODE provide foundational data for genome analysis.

**TABLE 7:** Common genomic reference models and their functions.

Model name	Function
GRCh38	A complete reference for the human genome.
ENCODE	Helps study how genes are regulated.
1000 Genomes Project	Provides data on genetic variations in humans.
RNAcmap	Helps understand RNA evolution.
BV-BRC	Contains information on bacterial and viral pathogens.

### 5.4 Encoder–decoder architectures in genomics

The encoder–decoder framework represents an efficient method for converting multiple genomic forms, combining the strengths of transformers and encoders with the advantages offered by decoders. This architecture, fundamentally used in machine translation and certain NLP tasks, is applied only to genomic problems of a narrow, specialized scope.

#### 5.4.1 Encoder–decoder model applications in genomics

Sequence-to-sequence tasks: For some genomics-related tasks, such as changing only one sequence into another, the use of an encoder–decoder model is very important.

For example, the model of predicting RNA secondary structure from a DNA sequence uses the encoder–decoder concept, which converts the input sequences to the structured outputs.

Multi-omic data integration: Given a variety of data types available, an encoder–decoder model functions as a tool to combine different types. A typical genomic sequence to epigenomic signals of DNA can be used to predict the regulatory activity of a sequence.

Variant-to-phenotype mapping: By establishing the correspondence between the gene mutations and the resulting traits, deep learning models can be used in the selection of targeted therapy and disease diagnosis. As shown in Table 8, encoder, decoder, and encoder–decoder architectures play distinct roles in genomic sequence modeling, each with specialized functions, strengths, and applications.

**TABLE 8:** Comparative overview of encoder, decoder, and encoder–decoder architectures in genomic modeling.

Type	Purpose	Main function	How it works?	Key strength	Common application	Example use cases in genomics	Example model
Encoder	Extracts features from the input	Analyzes and understands DNA/RNA sequences	Masks parts of sequences and predicts missing information	Excellent at identifying patterns, mutations, and functional elements in DNA	Promoter prediction, transcription factor binding site analysis, and single-nucleotide variation detection	Classification tasks (e.g., promoter identification)	DNABERT, Nucleotide-Transformer, GenFormer, and Uni-RNA
Decoder	Generates output sequences	Generates or predicts genetic sequences	Uses learned patterns to generate sequences	Strong generative ability for new sequences and evolutionary predictions	Generating new sequences, species identification, and regulatory factor prediction	Sequence reconstruction or prediction	Orca, GenSLMs, DNAGPT, HyenaDNA, and Evo
Encoder–decoder	Maps input to different output formats	Both analyzes and generates sequences	First encodes the sequence and then decodes it to reconstruct or predict	Combines analysis and generation for better results	Complex genomic tasks like sequence reconstruction and mutation prediction	Predicting 3D genome structure and chromatin interactions	Orca, C.Origami, and ENBED (Ensemble Nucleotide Byte-level Encoder–Decoder)

## 6 Datasets used for training Gene-LLMs

**FIGURE 8:**
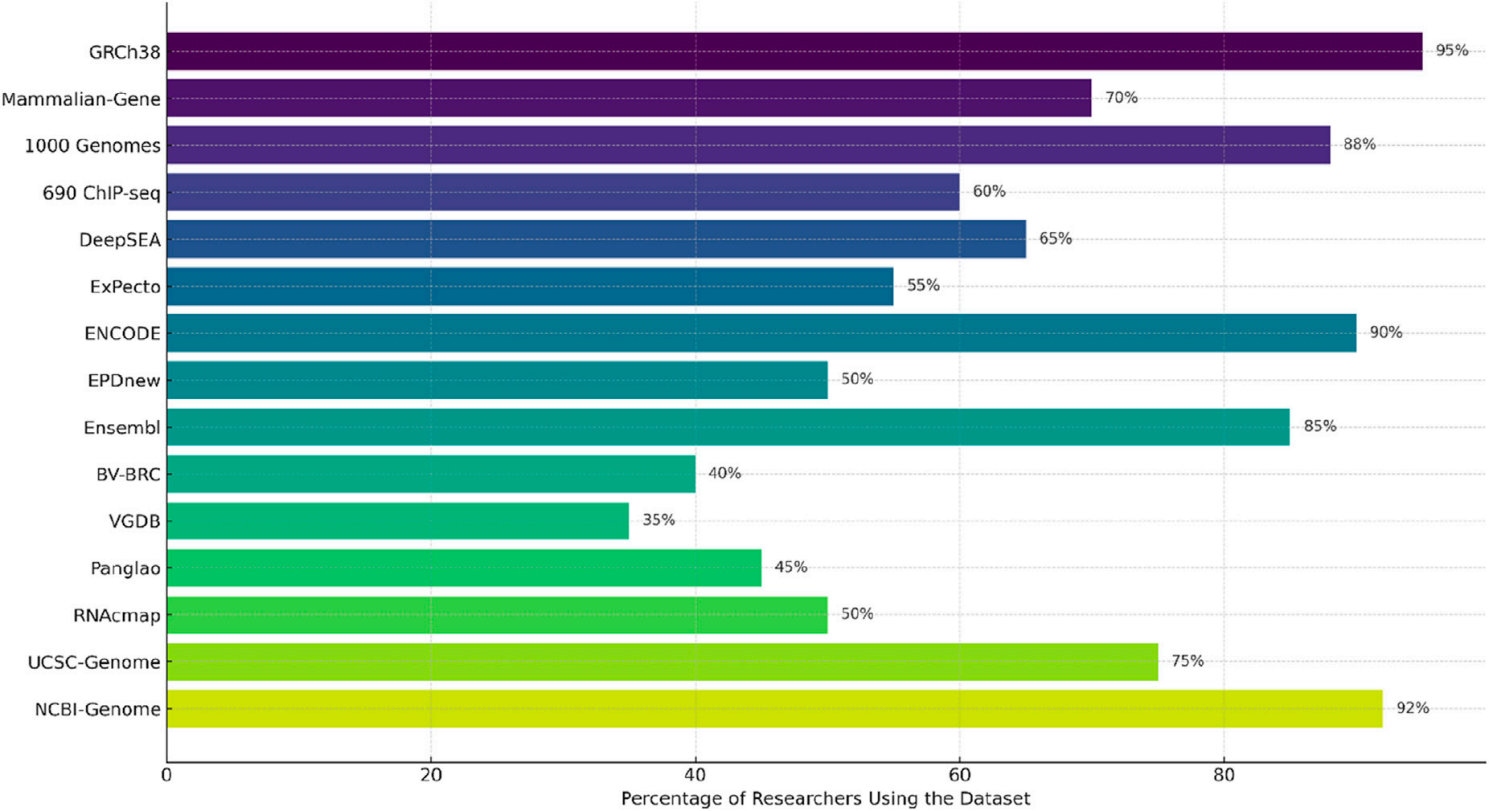
Usage distribution of genomic datasets across recent Gene LLM studies (2021–2025). Values represent relative usage frequency calculated based on citation counts, dataset mentions, and usage across selected genomic language model publications. A combination of literature survey and metadata extraction was used to estimate frequency.

Gene-LLMs necessitate a large amount of genome data for training. Genomic data from humans and other species are the data source of choice for scientists to improve the understanding of these models of DNA sequences. The main source of data is human and mammalian genomes. The GRCh38 dataset is used for training complete human DNA reference models. A complete description of the Mammalian-Gene dataset is provided in the study, which contains genetic information from mammals like chimpanzees and pigs; the 1,000 Genomes Project was a project that collected DNA from 2,500 people worldwide with the aim of studying diversity and variations in genetics. In the area of gene regulation and expression, datasets like 690 ChIP-seq provide insights into how proteins interact with DNA, thereby enabling models to gain a better understanding of gene activity. DeepSEA investigates DNA modifications and how they can affect gene function, but ExPecto tries to find an answer to the question of how different sequences of DNA affect gene expression across the tissues. One of the tasks of a functional genomics database is creating a genomic “atlas” that maps the regulatory elements of the genome. ENCODE is a superb dataset that is a landmark in terms of its breadth as it identifies the major functional regions of the human genome and even those that are not only genes and regulatory DNA sequences. The EPD new database remains the primary resource for identifying sequences that activate gene expression. In addition to this, Ensemble also has a big collection of data on the general biology of different species. Genomic and viral DNA are also quite significant in the training of Gene-LLMs. STAARS was one of the projects included in NPR, aiming to generate the first representative agent of the American BV-BRC, which stores both viral and bacterial DNA. However, among the DNA they collect, there are also various COVID-19 variants. On the other hand, VGDB is the viral genome collection that is mainly used for the identification of virus evolution and functioning. Many niche datasets are available to solve certain problems in genomic research. Panglao is specifically devoted to single-cell RNA data for studies on gene expression in different cell types. RNAcmap, in turn, uses evolutionary data to predict RNA structures. UCSC Genome is a very useful tool for sequence comparison across species, and the NCBI-Genome offers a multitude of genetic sequence records from various organisms that can be a support to any and all of the genomic research areas. From Figure 8, it is quite clear that datasets like GRCh38, ENCODE, and NCBI Genome are driving the development of Gene-LLMs by research scientists for general research usage. On the other hand, RNAcmap and Panglao are two different types of databases—RNAcmap is an RNA sequence repository, and Panglao is a single-cell gene database—which are used for Gene-LLM development with a narrower scope.

We manually examined peer-reviewed publications and preprints from 2021 to 2025 that either proposed or evaluated genomic language models to calculate the numbers represented in Figure 8. For each study, we documented the datasets used, extracted citation and usage metrics (if accessible), and normalized them to approximate relative frequency. Although this method may not account for all usages, it is indicative of the typical usage frequencies of the most cited and popular Gene-LLMs.

## 7 Benchmarks

Benchmarks are an essential part of the process of evaluating and comparing how different models can perform in genomic research. They help reveal the potential of Gene-LLMs in different genomic tasks. The following is a full description of the benchmarks that are most frequently used.

CAGI5 Challenge Benchmark (CAGI5) was created to assess the extent to which computers can replicate human ability in recognizing genetic alterations and their consequences. In cancer research, the goal is not to measure the overall impact of 17,500 DNA mutations but to understand how these mutations change genes and increase their susceptible to cancer. This is performed using a technology called the saturation mutagenesis massively parallel reporter assay. It examines a large number of mutations at the same time and identifies those that affect genotype–phenotype. The task also changes the protein during gene expression through exon splicing (a process that processes the gene to obtain further mRNA for gene phenotypic expression), introducing mutations that can disrupt protein functionality. In addition, it also checks model predictions for genetic mutations and their effect on the patient’s condition. In addition, the latter is considered clinical genome matching, which involves associating the model with real patient symptoms and complex genetic diseases, including intellectual disabilities and autism spectrum disorders, for diagnostic purposes.

The protein–RNA interaction prediction benchmark (protein–RNA) is designed to assess how accurately computational models can predict the interactions between proteins and RNA, which play a vital role in regulating gene expression and cellular function. Many machine learning (ML) models are designed for RNA–protein interactions, yet they typically use different training/evaluation datasets; therefore, the comparability of the work is compromised. This challenge, however, makes the comparison clear by introducing 37 different RBP interaction prediction models as the standard in the field. This is achieved using data from three leading CLIP-seq data centers, which together hold 313 different experiments involving 203 RBPs providing researchers with data for control experiments on RNA interactions.

The Nucleotide Transformer Benchmark (NT-Bench) allows new genomic models that focus on the nucleotide level to be measured qualitatively against foundational non-genomic models. This study worked on showing that the Knight–Beyer model is efficient in contrast to other reference models by performing eight different genomic tasks that are benchmarked by the former. Nucleotide Transformer is one of the tested models, while other models like DNABERT (a gene regulation-relevant adaptation of BERT), HyenaDNA (a predictive model that is optimized for long-distance genomic DNA data with context lengths 1 kb and 32 kb), and Enformer (a model with the ability of a deep learning mechanism to predict gene regulation) have been selected. This suggests that the former actually measured the models’ capability to perform the 18 tasks; accordingly, NNTBench also evaluated whether they integrate new data sources. The GenBench: A Benchmarking Suite for the Genomic Foundation Models (GFMs) cleaned the slate of all of the previous methods of evaluation in genomic research, particularly those on foundation models. This implies that it is well-versed in the methods and standards, comparable to those adopted by other researchers. A fair comparison is possible with GenBench through the standardization of experimental conditions, model complexity, dataset selection, and reproducibility. It is evident that the former method compared the two models for each of the 12 tasks. In the long-range genomic tasks, it was found that DNABERT, HyenaDNA, and Nucleotide Transformer were proven to be the best, while short-range genomic tasks showed HyenaDNA, Enformer, and DNABERT as the best. The 43 datasets, therefore, must be studied carefully to be able to compare the strengths and weaknesses of different models and address areas that need improvement.

**FIGURE 9:**
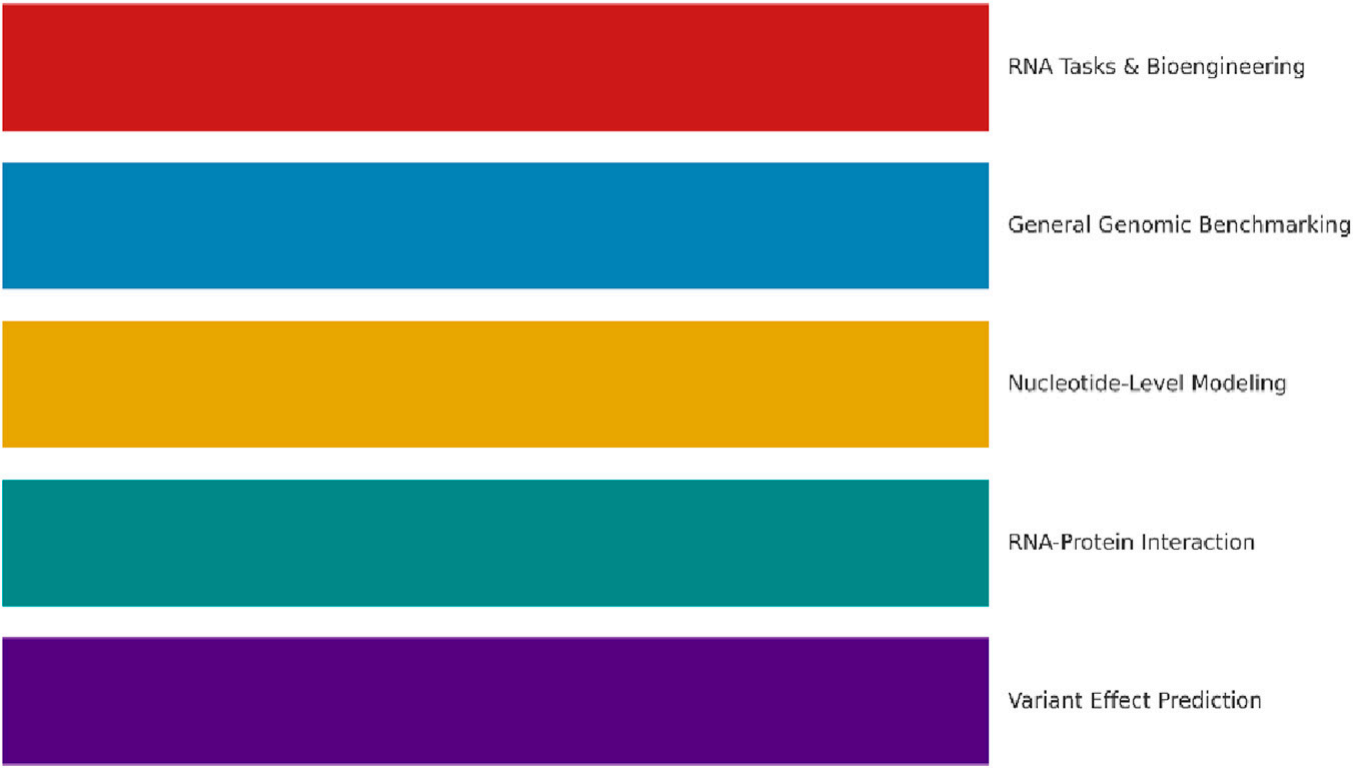
Mapping between representative genomic tasks and Gene-LLMs evaluated on them. This matrix highlights the relationship between language models and downstream genomic tasks, such as variant effect prediction, regulatory region classification, and gene expression modeling. The figure is intended to reveal patterns in model–task alignment and identify gaps in current benchmarking efforts.

The BEACON: Benchmark for RNA Language Models evaluates models’ performance in various RNA-related tasks, including structural, functional, and engineering challenges. By unraveling the mysteries of the RNA language, these models delve more deeply into RNA biology, creating a whole bunch of innovative RNA-based technologies that will significantly change the application scenarios in biomedicine, pharmaceuticals, agriculture, etc. Most of the projects discussed so far are focused on the medical field, but not all the papers’ innovations meet the same needs. In particular, innovation in the farm-to-table processes (crops) has been the industry’s main agenda. In cases where respondents answer affirmatively, training tests are required. Figure 9 depicts the connection between the variety of training datasets and the generalization performance of Gene-LLMs across benchmark tasks. The purpose of this figure is to demonstrate the extent to which diversified, heterogeneous datasets can enhance a model’s flexibility in handling different genomic tasks.

As shown in Figure 9, only a few tasks, such as variant effect prediction and promoter activity classification, have been cross-checked across multiple Gene-LLMs, while a large number of tasks have been only partially explored or not addressed at all. These mappings provide an idea of the places where the evaluation has been focused and the tasks to be covered for further work.

## 8 Evaluation of Gene-LLMs

Gene-LLMs are tested on four major tasks, namely, function prediction, structure prediction, sequence generation, and sequence variation and evolution analysis. These tasks allow scientists to reveal gene functions, understand molecular structures, create synthetic sequences, and explore genetic evolution. The function prediction task involves identifying gene functions and also studying the expression and regulation of the genes. Conventional models needed to be tailored to the specific task and then trained, but Gene-LLMs operate in a completely different way; they first acquire general knowledge from large genomic datasets and are then fine-tuned for a specific problem, thereby improving accuracy.

One of the most significant sub-tasks is promoter prediction, which mainly involves locating genes through regions of DNA promoters of DNA. It is the promoter that enables the RNA polymerase to stick to the DNA. Gene-LLMs, such as DNABERT, are trained on a large collection of human promoters (TATA and non-TATA) to identify sequence motifs, enabling more accurate repurposing of these sequences as promoters. The second subtask is enhancer prediction, and in this case, software is expected to locate the regions rich in enhancers, i.e., DNA sequences that are far from the genes but can regulate their transcription by attracting transcription-activating proteins. Although promoters are always next to the genes they control, enhancers can be located quite far away. Finding enhancers is of great importance to researchers for understanding the mechanism of gene regulation and disease. Gene-LLMS such as iEnhancer-BERT and iEnhancer-ELM are pre-trained on the human genome and later fine-tuned to discover enhancers.

Binding site prediction is the last part of the function prediction process, and its role is to find parts of the DNA, RNA, or protein that are the most probable contact points for molecules (such as transcription factors or other DNA- or RNA-binding proteins) to bind and act as controlling mechanisms for the expression of genes. The knowledge of binding sites is a prerequisite for gene regulation and drug discovery. New Gene-LLMs have been introduced as epigenomic gene prediction tools, e.g., EpiGePT, which scan DNA “bins” and scores sequences based on their potential correspondence with binding sites where proteins can attach, using DNA motifs and gene expression data to improve prediction accuracy. The process called structure prediction involves understanding the formation of helices and base pairs over time—in other words, the study of nucleic acid unfolding. This is the best model currently and is being applied in many such unpredictable cases in molecular biology. This allows for a better understanding of the gene-regulating process, the creation of new molecular devices, and the prediction of how mutations influence DNA/RNA structures in human diseases. One of the major steps in the chromatin profile prediction is identifying genes in DNA that are actively being expressed.

Chromatin is a complex of DNA and proteins that serves as the carrier of the genetic code. The packaging of genetic material within the core of the cell by chromatin prevents the free expression of genes. There are changes in the chromatin structure that affect gene expression and are associated with diseases like cancer. Understanding chromatin provides the potential to manipulate DNA indirectly, regulating gene activity without changing its structure. Gene-LLMs, such as HyenaDNA, are capable of predicting the locations of chromatin markers and epigenetic signatures from DNA sequences, which, in turn, aids in examining non-coding variants and their roles in gene regulation across the human genome. One of the subtasks is the DNA/RNA–protein interaction prediction task which aims to demonstrate the first possible way of recognizing a specific sequence of DNA within the protein sequence. This concurs with the interaction of DNA with the mRNA molecule itself. The regulation of both of these mechanisms is not only the result of a few disease-causing mutations but also a pathway for genetic defects that cause diseases. Multifaceted drug resistance (MDR) involves proteins with the ability to pump out many types of drugs, including anticancer drugs, resulting in resistance against chemotherapy treatments. Predicting protein-binding regions using TF-Bert from available DNA–protein interaction datasets offers a new approach that is valuable for gene regulation both now and in the future. The sequence generation task refers to a process where a model produces DNA code that resembles the real thing. This technique can be applied in cases such as genetic privacy, cost reduction in research, and improving data synthesis for model training purposes. Genome-LLMs (e.g., DNAGPT) are sources of artificial genomes that can be used to mask a person’s genetic features. DNAGPT has created 5,000 artificial genomes, each with 10,000 SNPs. These synthetic sequences were tested against real genetic data and outperformed other models in generating more realistic genetic variations. The sequence variation and evolution analysis task aims to study the process of DNA alteration throughout time and understand the impact of these DNA changes on diseases and how they determine characteristics. This process facilitates the detection of disease-related mutations in genes, identification of evolutionary changes in species, and a better understanding of genetic adaptation. The main model in the domain is GenSLMs, which use LLMs for genetic evolution analysis. The tool is particularly suitable for the study of the evolution of the SARS-CoV-2 genome, and to this end, it is being used to monitor viral mutations and changes and understand the evolution and spread of the virus to different geographical areas. GPN-MSA uses an innovative concept where the alignment of multiple genomes from different domains provides the basis for DNA language modeling. The model facilitates the process of sequence comparison, making it easy for geneticists to determine the evolutionary lineage of organisms using genome data. The core tasks and associated models used in Gene-LLMs are summarized in Table 9.

**TABLE 9:** Core genomic tasks addressed by Gene-LLMs with their subtasks, objectives, and representative models.

Primary task	Subtask	Purpose	Example model
Function prediction	Promoter prediction	Locate promoter regions to identify gene start sites	DNABERT
	Enhancer prediction	Detect distal DNA elements enhancing transcription	iEnhancer-BERT and iEnhancer-ELM
	Binding site prediction	Identify TF/protein binding regions	EpiGePT
Structure prediction	Chromatin profile prediction	Predict active DNA expression based on chromatin state	HyenaDNA
	DNA/RNA–protein interaction	Predict binding between nucleic acids and proteins	TF-BERT
Sequence generation	Synthetic genome generation	Create artificial genomes for simulation or data privacy	DNAGPT
Variation and evolution analysis	Mutation analysis	Understand mutational impact and evolution of species or viruses	GenSLMs
	Comparative genomics (MSA-based)	Align multiple genomes to analyze conserved sequences	GPN-MSA

**FIGURE 10:**
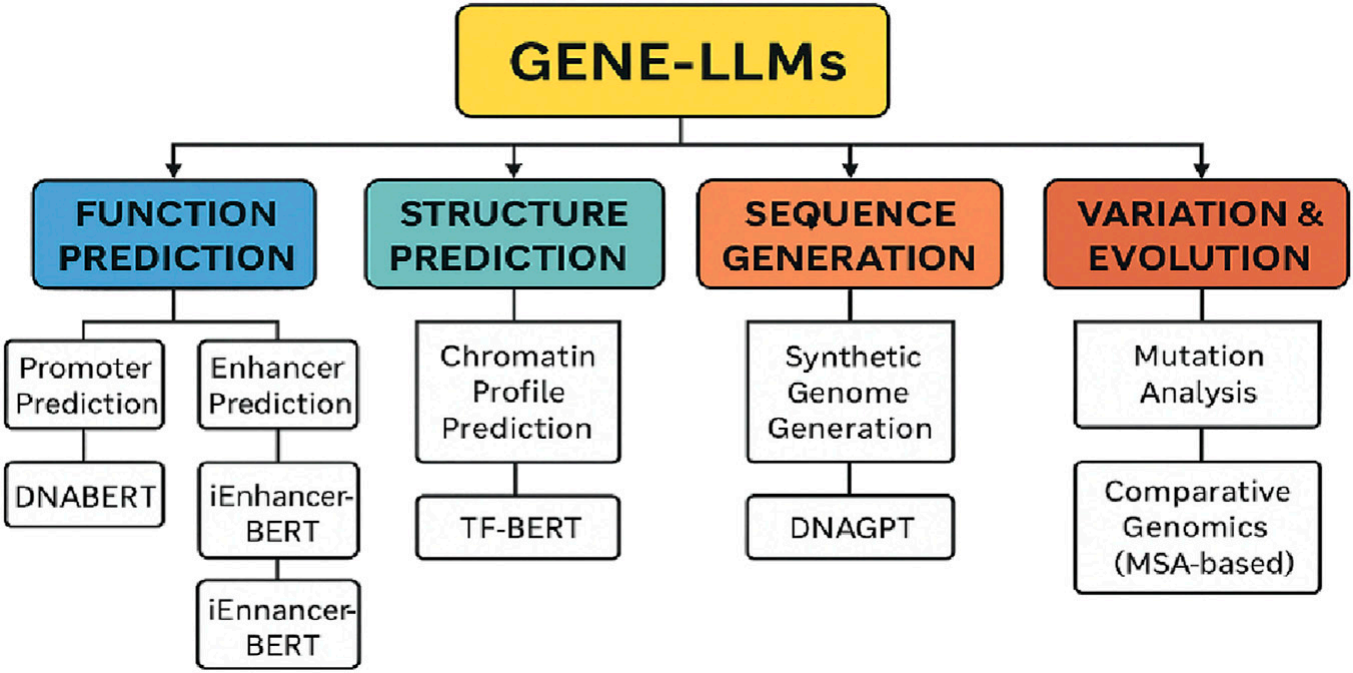
Categorization of core tasks performed by Gene-LLMs with associated subtasks and example models.

**FIGURE 11:**
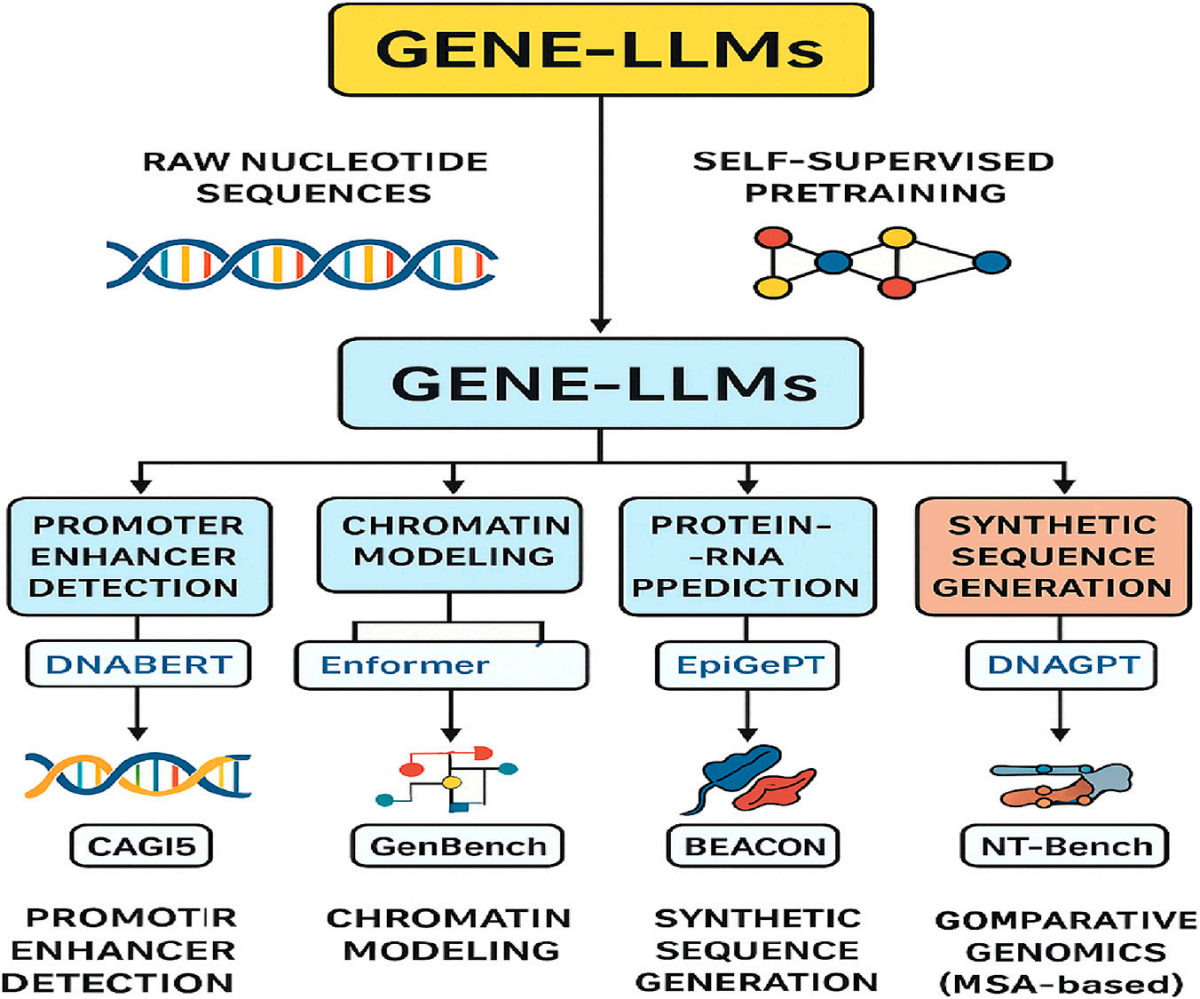
Workflow of Gene‐LLMs from raw nucleotide sequences to self-supervised pretraining, enabling key genomic tasks with example models and benchmarks.

Figure 10 shows the complexity of GPT or Gene-LLMs and how they are used in the four principal domains, namely, function prediction, structure prediction, sequence generation, and variation and evolution analysis, together with their corresponding subtasks and some model examples. The lifecycle of Gene-LLMs is provided in Figure 11.

### 8.1 Limitations and future directions

Despite the rapid advancements and promising potential of Gene-LLMs, several problems must be solved before their full application, especially in clinical and translational settings, can be realized. Moreover, recognizing these limitations is very important for guiding future research and ensuring responsible deployment.

#### 8.1.1 Data bias and representativeness

The datasets of genomic data available now often have biases in population representation, species diversity, and genomic region coverage. The lack of non-European ancestry in human datasets is just one example of how data bias can lead to uneven model performance across populations, potentially widening existing health disparities. On the other hand, bias in favor of model organisms (e.g., humans, mice, and yeast) restricts the transfer of knowledge from one organism to another. What new approaches could focus on selecting more diverse and balanced datasets, and how might synthetic data be used to supplement underrepresented genomic contexts?

#### 8.1.2 Interpretability and biological insight

Gene-LLMs are highly accurate in prediction; however, they often lack transparency in the steps leading to a certain decision, making it difficult to translate the results into human biological knowledge. The “black-box” nature of the method is a big obstacle to its use in clinical diagnostics, a high-stakes application. These XAI (explainable AI) methods, like attention map visualization, gradient-based attribution, and motif discovery tools act like as paleontologists digging for the regulatory grammar that these models have learned.

#### 8.1.3 Computational resource demands

Building a cutting-edge Gene-LLM is a huge computational task. It takes substantial GPU or TPU power, extensive storage, and long processing times, hours or even days. This is a major bottleneck for small laboratories that wish to do the same. However, one can reduce the resource demands through many approaches such as parameter-efficient fine-tuning (PEFT), model distillation, mixed-precision training, and cloud-based collaborative computing platforms, which can lower resource costs and broaden accessibility.

#### 8.1.4 Lack of robust clinical validation

Most of the Gene-LLM studies are basically confined to researching gene datasets, and only a small number of models have been put to the test in a clinical setting.

#### 8.1.5 Ethical, legal, and social implications

Genomic data are sensitive; hence, the issues of privacy, consent, and data sharing around the handling of genomic data are major concerns.

Gene-LLMs can revolutionize genomics and precision medicine as a whole; however, this transformation will require the community to first address a series of challenges, including data bias, interpretability of models, reduction of computational resources, clinical validation of models, and, finally, ethical issues. The community, by following these paths, is thereby committing to the development of Gene-LLMs as dependable, fair, and clinically implementable tools.

## 9 Conclusion

The era of Gene-LLMs has brought a revolution in computational genomics by connecting deep learning and molecular biology, making it possible to understand the language of life. Using transformer-based structures and self-supervised pretraining mechanisms—such as masked nucleotide modeling and sequence containment—Gene-LLMs generate informative representations from raw genetic sequences, gene expression profiles, and corresponding multi-omic annotations. This study is a complete Gene-LLMs life cycle from data ingestion and tokenization (k-mer and gene-level) to applications of the data, like protein–RNA interaction prediction and synthetic genome generation. The performance of the models is verified in several contexts: CAGI5, NT-Bench, GenBench, and BEACON for functional, structural, and evolutionary genomics. The harmonizing relationship between Gene-LLMs and WGS results in easy-to-understand clinical variant interpretation, which is further translated to precision diagnostics and targeted therapy. The most advanced encoder–decoder architectures, position-aware embeddings, and multimodal fusion strategies are being encompassed in studies of genomic representation learning to enable the incorporation of multiple data types in one model. Nonetheless, problems remain to be recognized, including the interpretability of the model, the scalability of computations, and the ethical governance of genomic data usage. In the future, breakthroughs in federated genomics learning, adaptation to the low-resource settings for rare variant detection, and bio-constrained pretraining will play a major role in realizing the full potential of Gene-LLMs. Nonetheless, Gene-LLMs are steadily establishing their role in the field of biomedicine, thus demonstrating a strong supportive ability in the sphere of individualized healthcare, drug discovery, and telling the story of evolution. There is an increasing need for ongoing collaboration among AI experts, molecular biologists, and clinicians in this field. Such collaboration offers three main benefits, namely, the manufacture of strong models that are both ethical and therefore safe (security in terms of the use of technology) and their effective application in promoting and accelerating genomic science.
